# Predicting MASLD in People With HIV Using Anthropometric Measures: A Multicenter Cross-Sectional Study

**DOI:** 10.1093/ofid/ofag236

**Published:** 2026-04-21

**Authors:** Jihad Slim, Murad Qirem, Paul Bellafiore, Bereket Tewoldemedhin, Tala B Shahin, Kevin Leyden, Ronald Poblete, Sandhya Nagarakanti, Hugo Vargas

**Affiliations:** Department of Infectious Diseases, NewYork Medical College, Valhalla, New York, USA; Department of Gastroenterology, New York Medical College, Valhalla, New York, USA; Department of Gastroenterology, New York Medical College, Valhalla, New York, USA; Department of Infectious Diseases, NewYork Medical College, Valhalla, New York, USA; Department of Internal Medicine, Mayo Clinic, Phoenix, Arizona, USA; Department of Infectious Diseases, North Jersey Community Research Initiative (NJCRI), Newark, New Jersey, USA; Department of Infectious Diseases, North Jersey Community Research Initiative (NJCRI), Newark, New Jersey, USA; Division of Infectious Diseases, Mayo Clinic, Phoenix, Arizona, USA; Division of Gastroenterology & Hepatology, Mayo Clinic, Phoenix, Arizona, USA

**Keywords:** MASLD, HIV, Anthropometric, Metabolic dysfunction-associated steatotic liver disease, Human Immunodeficiency Virus

## Abstract

**Background:**

Metabolic dysfunction–associated steatotic liver disease (MASLD) is increasingly recognized among people with human immunodeficiency virus (PWH), which is likely related to metabolic alterations and fat redistribution. Anthropometric measures such as body mass index (BMI) and waist circumference (WC) are commonly used for assessing metabolic risk. However, their predictive accuracy for MASLD in this population remains uncertain.

**Method:**

This multicenter cross-sectional study enrolled adults with HIV who underwent transient elastography with controlled attenuation parameter (CAP) measurement. Anthropometric and metabolic indices, including BMI, WC, hip circumference, waist-to-hip ratio (WHR), and visceral adiposity index (VAI), were collected from the study participants. Receiver operating characteristic (ROC) curve analyses were performed to determine the diagnostic performance of these indices for detecting hepatic steatosis, defined as CAP value of ≥248 dB/m. Analyses were stratified by sex assigned at birth.

**Results:**

Altogether, 256 participants were included. WC demonstrated the numerically highest discrimination for CAP-defined steatosis (area under the ROC curve 0.769), followed by BMI and hip circumference, whereas WHR and VAI showed weaker performance. In sex-stratified analyses, WC remained the numerically highest-performing measure among males, whereas BMI, WC, hip circumference, and VAI showed similar discrimination among females.

**Conclusions:**

Simple anthropometric measures, particularly WC, show fair ability to discriminate CAP-defined hepatic steatosis in PWH and may facilitate targeted fatty liver screening in routine HIV care.

Metabolic dysfunction–associated steatotic liver disease (MASLD) a highly prevalent chronic liver condition worldwide. Currently, ∼38% of adults and 7%–14% of children and adolescents are affected, and the prevalence among adults is projected to exceed 55% by 2040 [1]. MASLD has emerged as the most common chronic liver disease among people with human immunodeficiency virus (PWH), which is likely related to increased life expectancy and potential metabolic effects of antiretroviral therapy (ART) [2].

A recent meta-analysis by Wei et al. demonstrated that more than one-third of PWH have fatty liver disease, highlighting a substantial need for screening in this population [3]. HIV-specific factors such as chronic inflammation and long-term exposure to ART contribute to dysmetabolic changes, including lipodystrophy and hyperlipidemia. Additionally, metabolic risk factors, including obesity, type 2 diabetes mellitus, and hypertension, are more prevalent among PWH, further compounding MASLD risk [4, 5]. Consequently, PWH may develop hepatic steatosis at lower body mass index (BMI) levels, and the so-called “lean MASLD” is not uncommon in this group. The current American Association for the Study of Liver Diseases guidelines do not recommend universal MASLD screening in PWH, focusing instead on individuals with elevated aminotransferase levels or metabolic syndrome features [6]. Therefore, simple and readily available surrogates of liver fat could be valuable tools in HIV care to identify high-risk individuals who may benefit from further evaluation [2, 3].

MASLD is defined by the presence of hepatic steatosis on imaging or histology, along with at least one metabolic abnormality (eg, obesity or increased waist circumference (WC), type 2 diabetes mellitus, hypertriglyceridemia, low high-density lipoprotein [HDL] cholesterol, or hypertension), in the absence of marked alcohol consumption [2]. Controlled attenuation parameter (CAP) measured by transient elastography (FibroScan) is a widely used noninvasive method to quantify liver fat with a good diagnostic accuracy [3, 7]. The CAP values correlate with steatosis grade on imaging and histology; a CAP threshold of ≥248 dB/m is commonly used to indicate at least mild hepatic steatosis [4, 8]. Although elastography offers a practical alternative to liver biopsy, its availability remains limited in some clinical settings. Therefore, it is clinically relevant to determine whether simple anthropometric measures could serve as predictors of liver fat accumulation in PWH to identify individuals at a higher risk of MASLD who may benefit from further evaluation using routine clinical data.

Central obesity is a key driver of MASLD pathogenesis. WC is a simple measure of central adiposity that correlates closely with visceral fat and metabolic risk and is included as a criterion for metabolic syndrome and MASLD [2]. BMI, although widely used to determine obesity, does not capture fat distribution, yet it remains consistently associated with MASLD risk [9]. Hip circumference reflects gluteofemoral fat, which is generally considered metabolically less harmful than visceral fat, and its relationship with hepatic steatosis is less well established. The waist-to-hip ratio (WHR) integrates both waist and hip measurements and reportedly predicts MASLD and liver-related outcomes in some populations [10]. Additionally, composite indices, including the visceral adiposity index (VAI)—which incorporates WC, BMI, triglycerides, and HDL cholesterol—have been developed to estimate visceral fat burden [10]. Individuals with MASLD tend to have higher VAI values than controls, and VAI increases with greater steatosis severity [11–14]. Given that hypertriglyceridemia is a prominent feature of HIV-associated MASLD [13], VAI may be a potentially useful marker in this population. However, it remains unclear which measure—simple anthropometric indices or VAI—best correlates with hepatic steatosis in PWH.

The present study aimed to determine which anthropometric measure correlates most strongly with liver fat accumulation in PWH, as reflected by the CAP score. In this study, we performed a multicenter cross-sectional analysis comparing WC, hip circumference, BMI, WHR, and VAI against the CAP values and evaluated their ability to predict significant steatosis through a receiver operating characteristic (ROC) curve analysis.

## METHODS

### Study Design and Population

This multicenter cross-sectional study enrolled adult PWH from 2 tertiary HIV clinics in Newark, New Jersey. The participants were recruited consecutively during routine clinic visits between 2022 and 2024. Eligible individuals were aged ≥18 years, had documented HIV infection on stable ART with a plasma HIV viral load of <200 copies/mL, and had no active hepatitis B or C infection. Participants with positive hepatitis B surface antigen or detectable hepatitis C RNA were excluded from the analysis. Stable ART was defined as the receipt of the same antiretroviral regimen for at least 12 months prior to enrollment. Hepatitis B surface antigen and hepatitis C RNA testing were performed within 3 months of elastography.

Participants with excessive alcohol use or other chronic liver diseases were also excluded from the analysis. Excessive alcohol use was defined as an alcohol use disorders identification test–consumption (AUDIT-C) score of ≥4 and ≥3 for men and women, respectively. The other chronic liver diseases excluded included autoimmune hepatitis, hemochromatosis, Wilson disease, alpha-1 antitrypsin deficiency, and medication-associated liver disease, based on chart review.

All eligible clinic patients during the study period were invited to participate and given an equal opportunity to enroll in the study. No additional prescreening or risk-based selection was used beyond the predefined inclusion and exclusion criteria. Each site obtained Institutional Review Board approval, and all participants provided written informed consent for study participation.

### Data Collection

We recorded the participants' demographics (age, sex assigned at birth, race, and ethnicity) and clinical factors, including current ART regimen and comorbidities. Diabetes mellitus was defined as hemoglobin A1c of ≥6.5%, fasting plasma glucose level of ≥126 mg/dL, random plasma glucose level of ≥200 mg/dL, or use of glucose-lowering drugs. Hypertension was defined as a systolic blood pressure of ≥130 mmHg, diastolic blood pressure of ≥80 mmHg, or use of antihypertensive drugs. Dyslipidemia was defined as a total cholesterol level of ≥200 mg/dL, LDL-cholesterol level of ≥130 mg/dL, HDL-cholesterol level of <40 mg/dL in men and <50 mg/dL in women, triglyceride level of ≥150 mg/dL, or use of lipid-lowering drugs. Concomitant medications and alcohol intake (using the AUDIT-C questionnaire) were recorded. CD4 cell count and plasma HIV viral load were obtained within 3 months of the elastography.

Anthropometric measurements were obtained by trained staff using standardized techniques. WC was measured at the midpoint between the lowest rib and iliac crest, and hip circumference at the widest part of the hips; both were recorded to the nearest 0.1 cm. Height and weight were measured to calculate BMI (kg/m^2^). WHR was then derived as WC divided by hip circumference. VAI was also obtained for each participant using Amato et al.'s formula, which incorporates WC, BMI, triglycerides, and HDL cholesterol using sex-specific constants [15]. Fasting blood samples collected within 3 months of the anthropometric measures provided data on triglycerides, HDL cholesterol, and other laboratory parameters needed for VAI and metabolic assessments.

### Liver Fat Assessment

Each participant underwent elastography with CAP (FibroScan) to noninvasively quantify hepatic steatosis. FibroScan examinations were performed using Echosens FibroScan 230. M or XL probes were used according to manufacturer recommendations based on body habitus. Examinations were performed after at least 3 hours of fasting. Ten valid measurements were acquired, and the median CAP value (in decibels per meter, dB/m) was recorded along with the interquartile range/median (IQR/M) to ensure measurement reliability (IQR/M, <30%). The CAP value reflects ultrasound attenuation due to liver fat, with higher values indicating greater steatosis. We defined significant steatosis as a CAP value of ≥248 dB/m, a cutoff corresponding to ≥S1 (≥11% liver fat) based on prior validation studies [8]. Liver stiffness (FibroScan kPa) was also measured for fibrosis assessment, but the present analysis focused on steatosis.

### Statistical Analysis

The participants' baseline characteristics were summarized as mean ± standard deviation for continuous variables and counts (percentages) for categorical variables. We conducted an ROC curve analysis and calculated the area under the ROC curve (AUC) for WC, hip circumference, BMI, WHR, and VAI. The AUC value was compared with the 0.50 no-discrimination line, with values closer to 1.0 indicating better discrimination. By convention, we considered an AUC of >0.8 as good, 0.7–0.8 as fair, 0.6–0.7 as poor, and 0.5–0.6 as failing to discriminate. The optimal cutoff values for each predictor were identified by maximizing the Youden index (sensitivity + specificity—1). We constructed the ROC curves with 95% confidence intervals (CIs) for visual comparison. Statistical analyses were performed using SPSS. We repeated the ROC curve analyses stratified by sex assigned at birth to examine the potential sex-specific differences. Missing data were handled through a complete-case analysis. Missingness was <5% for all variables.

## RESULTS

### Baseline Characteristics

During the study period, 327 individuals were screened for eligibility. 71 participants were excluded due to excessive alcohol consumption or the presence of other chronic liver diseases. We analyzed the data from 256 PWH with a mean age of 53.97 ± 13.72 years (Table 1). Approximately two-thirds of the participants were male (68.8%); 57% identified as black, 42% as white, <1% as Asian, and one-third (33.6%) as Hispanic. The cohort had a high prevalence of metabolic comorbidities. The mean BMI was 29.41 ± 6.28 kg/m^2^, placing the average participant in the overweight range, and nearly 40% met the BMI criteria for obesity. Central adiposity was notable, with a mean WC of 98.7 ± 19.2 cm. Over half of the patients (52.4%) had hypertension, and 17.7% had type 2 diabetes mellitus. The mean hip circumference and WHR were 104.2 ± 13.2 cm and 0.95 ± 0.19, respectively. The laboratory results indicated well-controlled HIV infections in most participants (mean CD4 count, 689 ± 286.55 cells/µL), and the majority had undetectable viral loads, with some degree of dyslipidemia (38% were on active treatment). The VAI values ranged widely (mean, 4.42 ± 6.14).

**Table 1.** Characteristics of the Study Participants (N = 256)

Variable	Value
Age (years)	53.97 ± 13.72
Sex, n (%)	Males 176 (68.75%)
	Females 80 (31.25%)
Race, n (%)	Black 146 (57.0%)
	White 108 (42.2%)
	Asian 2 (0.8%)
Ethnicity, n (%)	Non-Hispanic 170 (66.4%)
	Hispanic 86 (33.6%)
Weight (kg)	86.45 ± 20.55
Height (m)	1.71 ± 0.10
Body mass index (kg/m^2^)	29.41 ± 6.28
Waist circumference (cm)	98.72 ± 16.17
Hip circumference (cm)	104.20 ± 13.1
Waist-to-hip ratio	0.95 ± 0.16
Hypertension, n (%)	119 (52.42%)
Triglycerides (mg/dL)	121.86 ± 66.23
Diabetes mellitus, n (%)	42 (17.72%)
HDL cholesterol (mg/dL)	51.16 ± 15.99
LDL cholesterol (mg/dL)	103.37 ± 36.04
Total cholesterol (mg/dL)	174.33 ± 40.49
On dyslipidemia therapy, n (%)	98 (38.3%)
On diabetes therapy, n (%)	24 (9.4%)
CD4 count (cells/µL)	689.36 ± 286.55
FIB-4 score (units)	1.37 ± 1.08
CAP score (dB/m)	244.81 ± 57.48
Visceral adiposity index	4.42 ± 6.14

Hepatic steatosis was common in this cohort. The mean CAP value was 244.8 ± 57.5 dB/m, and 110 participants (43.0%) had CAP of ≥248 dB/m, which was consistent with at least mild steatosis. The remaining 57% had CAP values below this threshold. Thus, roughly 4 in 10 PWH in our sample met the criteria for liver steatosis, highlighting a substantial burden of CAP-defined steatosis in this population.

### Diagnostic Performance (ROC Analysis)

**Figure 1.**
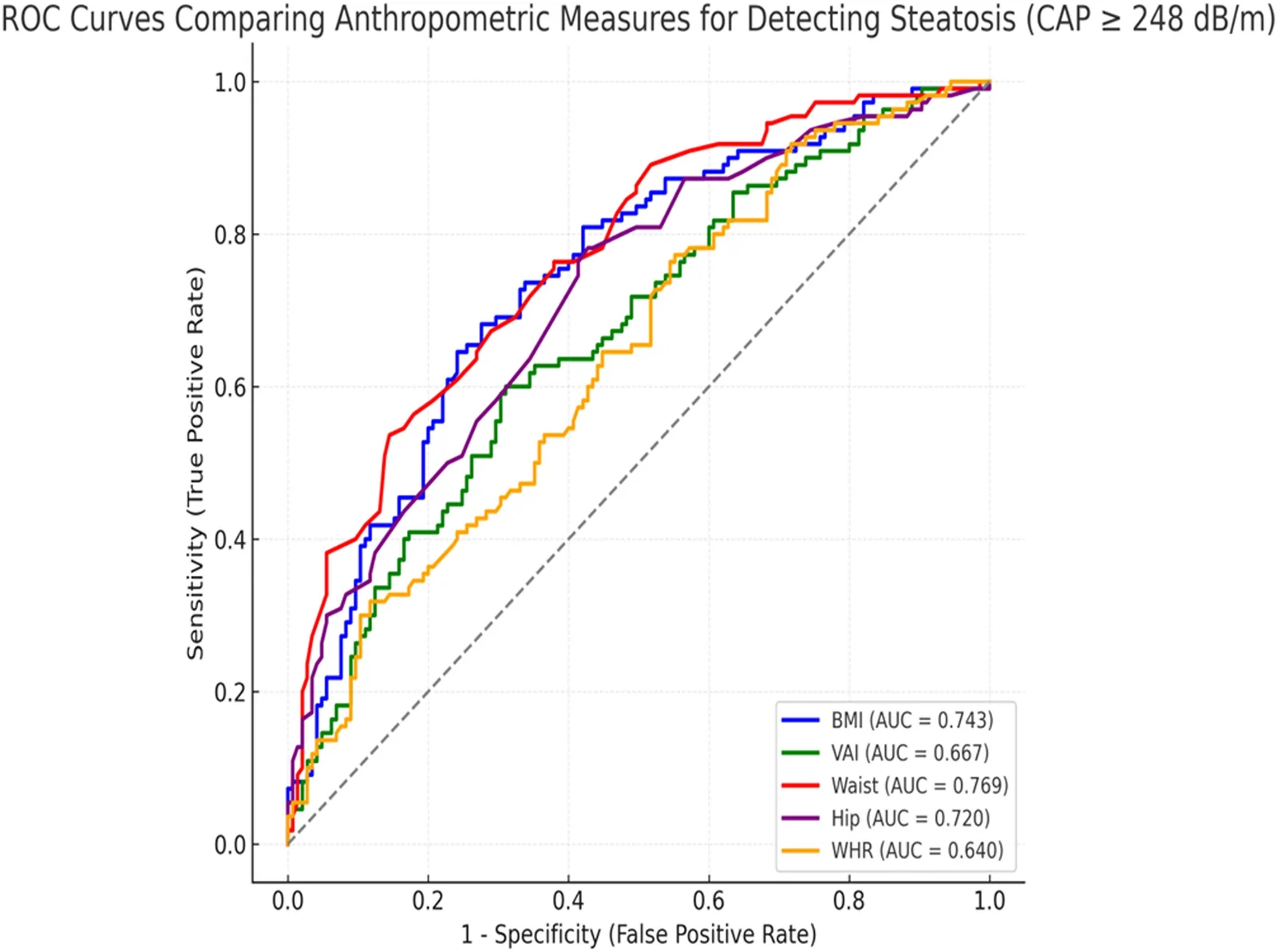
ROC curves comparing anthropometric measures for detecting significant steatosis (CAP ≥248 dB/m). ROC curves comparing the discriminative performance of BMI, VAI, waist circumference, hip circumference, and WHR in predicting significant hepatic steatosis (CAP ≥248 dB/m).

We examined the discriminatory performance for significant steatosis (CAP, ≥248 dB/m) (Figure 1). WC demonstrated the highest discriminatory ability (AUC 0.769, 95% CI: 0.709–0.829). At an optimal cutoff of 106 cm, WC yielded 53.6% sensitivity and 85.5% specificity for detecting steatosis (Table 2). BMI showed a fair diagnostic performance (AUC 0.743, 95% CI: 0.681–0.805); a BMI cutoff of 29.1 kg/m^2^ yielded 68.2% sensitivity and 72.4% specificity. Hip circumference had an AUC of 0.720 (95% CI: 0.656–0.785). VAI demonstrated a lower performance (AUC 0.667, 95% CI: 0.599–0.734). WHR had the lowest AUC value (0.640, 95% CI: 0.562–0.718). All predictors had AUC values significantly >0.50 (*P* < .001 for each), suggesting that each measure carried some information for steatosis detection, although the prediction strength varied.

**Table 2.** ROC Analysis of Anthropometric Measures for Detecting Significant Steatosis (CAP ≥248 dB/m)

Measure	AUC (95% CI)	Optimal Cutoff	Sensitivity at the Cutoff (%)	Specificity at the Cutoff (%)
Waist circumference (cm)	0.769 (0.709–0.829)	106.0	53.6	85.5
BMI	0.743 (0.681–0.805)	29.09	68.2	72.4
Hip circumference (cm)	0.720 (0.656–0.785)	101.0	78.2	57.2
Visceral adiposity index	0.667 (0.599–0.734)	3.82	60.0	69.0
Waist-to-hip ratio	0.640 (0.562–0.718)	0.91	77.3	44.8

This table summarizes the diagnostic performance of anthropometric measures for predicting significant hepatic steatosis (defined as CAP ≥248 dB/m). The area under the ROC curve (AUC) with 95% confidence intervals (CI), optimal cutoff (Youden index), and corresponding sensitivity and specificity values are presented.

### Sex-Stratified Analysis

Given the known sex differences in fat distribution, we conducted analyses stratified by sex (Table 3). Among the male participants (n = 176), WC remained the strongest predictor with the highest AUC value for steatosis detection (AUC 0.802, 95% CI: 0.734–0.871). Contrarily, in women (n = 80), the advantage of WC was less pronounced, with an AUC of 0.697 (95% CI: 0.579–0.814). In women, BMI, WC, and hip circumference showed comparable AUC values (∼0.70), indicating that hip circumference and BMI were as predictive of liver fat as WC in women. WHR showed sex-specific differences: in men, WHR correlated with CAP with an AUC of 0.709; and in women, WHR was not significantly associated with CAP as the ROC curve was essentially no better than chance (AUC = 0.526, 95% CI: 0.398–0.654). VAI was somewhat more predictive in women (AUC, 0.712) than in men (AUC, 0.648), although its performance remained lower than those of the basic anthropometric measures.

**Table 3.** Sex-Stratified ROC Curve Analysis of Anthropometric Measures for Detecting Significant Steatosis (CAP ≥248 dB/m)

Measure	Males AUC (95% CI)	Females AUC (95% CI)
BMI	0.756 (0.682–0.831)	0.718 (0.604–0.833)
VAI	0.648 (0.565–0.732)	0.712 (0.597–0.827)
WC	0.802 (0.734–0.871)	0.697 (0.579–0.814)
Hip circumference	0.740 (0.664–0.817)	0.704 (0.587–0.820)
WHR	0.709 (0.630–0.788)	0.526 (0.398–0.654)

## DISCUSSION

In this multicenter study involving PWH, WC showed the numerically highest discriminatory ability for CAP-defined hepatic steatosis compared with BMI, hip circumference, WHR, and VAI. WC had the highest AUC and fair diagnostic performance for identifying participants meeting the study definition of steatosis. These findings underscore the role of central adiposity in the pathogenesis of steatosis among PWH. Contrarily, VAI—despite incorporating lipid parameters—showed only a modest association with hepatic steatosis and limited discriminatory ability. This suggests that, in this cohort, simple WC measurement provided more clinically actionable information compared with a composite index.

Our findings are consistent with prior observations in the general population and among PWH, in whom central obesity is a key driver of MASLD [3, 9]. Visceral adipose tissue is metabolically active and can contribute to liver fat accumulation and insulin resistance through increased free fatty acid flux and inflammatory mediators to the portal circulation [16]. Accordingly, WC—a proxy for visceral adiposity—correlated strongly with liver fat. In a meta-analysis of MASLD risk factors in PWH, higher BMI and WC were associated with an increased risk of steatotic liver disease [3]. Our study reinforces these findings by quantifying the diagnostic performance and showing that WC demonstrated the highest discriminatory ability for steatosis among the anthropometric measures. This aligns with the MASLD criteria that include elevated WC as a metabolic risk factor [17]. Notably, WC approached the performance of some fatty liver-specific indices developed for the general population, and it is similar to the accuracy of the fatty liver index (FLI) reported in other studies; FLI is another composite score that includes WC, but our data suggest that WC itself carries much of the signal [18].

BMI also showed fair discrimination, although lower than that of WC. BMI does not capture fat distribution and can be less informative in settings characterized by fat redistribution. PWH may experience body composition changes—for example, some may experience relative preservation of peripheral fat with accumulation of visceral fat [3]. In such scenarios, 2 individuals with the same BMI could have very different liver fat risks depending on how much weight is carried centrally. This may explain why the correlation of BMC with CAP was less pronounced than that of WC. Nonetheless, BMI was still a useful marker (AUC 0.74), reflecting that overall adiposity does contribute to liver steatosis risk. Interestingly, in this cohort, hepatic steatosis was observed even among individuals who were not obese based on the BMI criteria, consistent with a prior study reporting that hepatic steatosis can occur at lower BMI values among PWH compared with HIV-negative populations [3]. This suggests that the BMI thresholds for MASLD risk might be lower in the context of HIV, and central fat measures, including WC, become particularly important for identifying at-risk individuals without obesity in this population.

Hip circumference showed fair discrimination. On face value, one might expect hip size (reflecting subcutaneous gluteofemoral fat) to be less relevant to liver steatosis. Indeed, gluteal fat is considered metabolically protective in some studies, and a higher hip circumference at a given WC is often associated with a better metabolic profile [19]. The observed relationship with CAP-defined steatosis likely arises because hip circumference increases in parallel with overall obesity, potentially acting as a general adiposity marker.

Some evidence suggests that WHR could outperform BMI in predicting liver-related outcomes [9, 20], as WHR captures fat distribution. However, in our study, WHR did not outperform other measures in the overall analyses and demonstrated clear sex-specific limitations. WHR had limited value among women, likely because the WC increases among women were often accompanied by increases in hip circumference, which can preserve the ratio despite the higher absolute adiposity. Contrarily, WHR performed better among men, consistent with known sex-based patterns of fat distribution [21]. A previous study suggested that men tend to accumulate adipose tissue centrally throughout their lifetime, whereas the women's fat distribution is heavily influenced by estrogen, resulting in a gluteofemoral fat pattern [21]. Furthermore, a meta-analysis by Balakrishnan et al. concluded that women have a lower risk of MASLD than men, largely due to their greater gluteofemoral fat deposition compared with the central fat predominance in men [22].

VAI was hypothesized to perform well given the importance of dyslipidemia and metabolic risk among PWH, yet it underperformed relative to the simple anthropometric measures. Several factors may explain this. First, VAI is derived from multiple variables and may be influenced by lipid-lowering therapy or other interventions that modify triglycerides and HDL-cholesterol independent of visceral fat burden. Additionally, VAI was developed in HIV-negative populations, and HIV-associated body composition changes (including lipoatrophy or redistribution of fat depots) may reduce its validity in PWH. WC reportedly can outperform VAI in predicting liver fat in some settings [23, 24]. In practical terms, these results suggest that the additional complexity of calculating VAI may not provide an incremental benefit over WC for steatosis screening in routine HIV care.

Clinically, WC is inexpensive, noninvasive, and feasible in routine practice. Our study findings suggest that PWH with elevated WC should be considered at a higher risk for MASLD and may benefit from targeted liver fat assessment or early lifestyle interventions. Reliance on BMI alone may underestimate the risk among individuals with normal BMI and central adiposity. Incorporating WC into routine HIV care may facilitate more efficient triage for elastography or other liver evaluation modalities.

### Strengths and Limitations

The present study benefits from a well-characterized multicenter cohort and the use of CAP, an objective measure of hepatic steatosis validated in MASLD [7]. We directly compared multiple indices side-by-side and performed sex-stratified analyses. However, several limitations should be acknowledged. First, the cross-sectional design only captures associations at one time point; thus, causality cannot be inferred; longitudinal studies are needed to see if WC increases can predict future steatosis or fibrosis progression in PWH.

Second, the VAI values varied widely and may have been influenced by treatment-related lipid changes, which could attenuate the correlations with CAP. Additionally, the use of a single-time-point lipid measurement may not fully reflect chronic visceral adiposity, further limiting the precision of VAI as a surrogate marker in this setting.

Third, CAP is an indirect measure of steatosis. Although we used a CAP threshold of ≥248 dB/m based on prior literature data [8], misclassification near the cutoff is possible.

Fourth, the cohort primarily included middle-aged PWH receiving contemporary ART, which may limit the generalizability of the study findings to other populations. Additionally, the number of female participants was smaller (n = 80), limiting the precision of sex-stratified estimates. Thus, these sex-specific findings should be interpreted with caution and warrant validation in larger cohorts of female PWH.

Finally, we did not fully characterize prior ART exposure. Although the current regimens were recorded, historical regimens were not consistently available, and cumulative exposure to older therapies may influence fat redistribution and steatosis risk. The HIV infection duration also could not be reliably determined, as many participants were unable to recall the exact time of diagnosis and medical records often lacked this information, representing potential unmeasured confounding.

## CONCLUSION

WC was the strongest anthropometric predictor of hepatic steatosis among PWH, demonstrating the highest correlation with CAP and the best diagnostic accuracy for identifying participants meeting the study definition of hepatic steatosis. BMI and hip circumference were also associated with hepatic steatosis, whereas VAI showed a more modest predictive performance. The results of the sex-stratified analyses suggest that WC is particularly informative among men, whereas BMI and hip circumference may be similarly informative among women, and WHR is not useful in women.

Integrating routine WC monitoring into HIV care could facilitate earlier identification of individuals at a higher risk of steatosis who may benefit from confirmatory liver evaluation. This could facilitate earlier management of modifiable risk factors and more targeted referral for elastography or other liver assessments. Future studies should evaluate whether the interventions that reduce central adiposity—through lifestyle changes or treatment optimization—are associated with the improvements in CAP and clinical outcomes among PWH.

## References

[1] Younossi ZM, Kalligeros M, Henry L. Epidemiology of metabolic dysfunction-associated steatotic liver disease. Clin Mol Hepatol 2025; 31:S32–50. 10.3350/cmh.2024.0431 PMC11925440 39159948

[2] Kalopitas G, Arvanitakis K, Tsachouridou O, et al Metabolic dysfunction-associated steatotic liver disease in people living with HIV-limitations on antiretroviral therapy selection. Life (Basel) 2024; 14:742. 10.3390/life14060742 PMC11205092 38929725

[3] Wei J, Hui W, Fang Y, et al The prevalence of nonalcoholic fatty liver disease in people living with HIV: a systematic review and meta-analysis. BMC Infect Dis 2025; 25:239. 10.1186/s12879-025-10455-y PMC11921747 40108499

[4] Arrive E, Viard JP, Salanave B, et al & ANRS CO19 COVERTE and ENNS study groups (20100). metabolic risk factors in young adults infected with HIV since childhood compared with the general population. PLoS One 2018;13:e0206745. 10.1371/journal.pone.0206745 PMC6226109 30408056

[5] Trachunthong D, Tipayamongkholgul M, Chumseng S, Darasawang W, Bundhamcharoen K. Burden of metabolic syndrome in the global adult HIV-infected population: a systematic review and meta-analysis. BMC Public Health 2024; 24:2657. 10.1186/s12889-024-20118-3 PMC11438355 39342258

[6] Rinella ME, Neuschwander-Tetri BA, Siddiqui MS, et al AASLD practice guidance on the clinical assessment and management of nonalcoholic fatty liver disease. Hepatology 2023; 77:1797–835. 10.1097/HEP.0000000000000323 PMC10735173 36727674

[7] Sardjan J, Lesmana CRA, Rusdi L, et al Correlation between controlled attenuation parameter values with SYNTAX score in patients with significant coronary artery disease. Sci Rep 2024; 14:15382. 10.1038/s41598-024-63792-4 PMC11224258 38965252

[8] Karlas T, Petroff D, Sasso M, et al Individual patient data meta-analysis of controlled attenuation parameter (CAP) technology for assessing steatosis. J Hepatol 2017; 66:1022–30. 10.1016/j.jhep.2016.12.022 28039099

[9] Yu C, He S, Kuang M, et al Association between weight-adjusted waist index and non-alcoholic fatty liver disease: a population-based study. BMC Endocr Disord 2024; 24:22. 10.1186/s12902-024-01554-z PMC10874525 38369482

[10] Åberg F, Färkkilä M, Salomaa V, et al Waist-hip ratio is superior to BMI in predicting liver-related outcomes and synergizes with harmful alcohol use. Commun Med (Lond) 2023; 3:119. 10.1038/s43856-023-00353-2 PMC10482890 37674006

[11] Ismaiel A, Jaaouani A, Leucuta DC, Popa SL, Dumitrascu DL. The visceral adiposity Index in non-alcoholic fatty liver disease and liver fibrosis-systematic review and meta-analysis. Biomedicines 2021; 9:1890. 10.3390/biomedicines9121890 PMC8698356 34944706

[12] Li Q, Wang L, Wu J, Wang J, Wang Y, Zeng X. Role of age, gender and ethnicity in the association between visceral adiposity index and non-alcoholic fatty liver disease among US adults (NHANES 2003-2018): cross-sectional study. BMJ open 2022; 12:e058517. 10.1136/bmjopen-2021-058517 PMC8938699 35314476

[13] Seth A, Sherman KE. Fatty liver disease in persons with HIV infection. Top Antivir Med 2019; 27:75–82. PMC6550355 31136997

[14] Yi X, Zhu S, Zhu L. Diagnostic accuracy of the visceral adiposity index in patients with metabolic-associated fatty liver disease: a meta-analysis. Lipids Health Dis 2022; 21:28. 10.1186/s12944-022-01636-8 PMC8898453 35249545

[15] Amato MC, Giordano C, Galia M, et al Visceral adiposity Index: a reliable indicator of visceral fat function associated with cardiometabolic risk. Diabetes care 2010; 33:920–2. 10.2337/dc09-1825 PMC2845052 20067971

[16] Bansal S, Vachher M, Arora T, Kumar B, Burman A. Visceral fat: a key mediator of NAFLD development and progression. Hum Nutr Metab 2023; 33:200210.

[17] Chan WK, Chuah KH, Rajaram RB, Lim LL, Ratnasingam J, Vethakkan SR. Metabolic dysfunction-associated steatotic liver disease (MASLD): a state-of-the-art review. J Obes Metab Syndr 2023; 32:197–213. 10.7570/jomes23052 PMC10583766 37700494

[18] Motamed N, Sohrabi M, Ajdarkosh H, et al Fatty liver index vs waist circumference for predicting non-alcoholic fatty liver disease. World J Gastroenterol 2016; 22:3023–30. 10.3748/wjg.v22.i10.3023 PMC4779925 26973398

[19] Stefan N . Causes, consequences, and treatment of metabolically unhealthy fat distribution. Lancet Diabetes Endocrinol 2020; 8:616–27. 10.1016/S2213-8587(20)30110-8 32559477

[20] Xie W, Hong Y, Chen X, Wang S, Zhang F, Chi X. Waist-to-hip ratio and nonalcoholic fatty liver disease: a clinical observational and Mendelian randomization analysis. Front Nutr 2024; 11:1426749. 10.3389/fnut.2024.1426749 PMC11563977 39555187

[21] Chang E, Varghese M, Singer K. Gender and sex differences in adipose tissue. Curr Diab Rep 2018; 18:69. 10.1007/s11892-018-1031-3 PMC6525964 30058013

[22] Balakrishnan M, Patel P, Dunn-Valadez S, et al Women have a lower risk of nonalcoholic fatty liver disease but a higher risk of progression vs men: a systematic review and meta-analysis. Clin Gastroenterol Hepatol 2021; 19:61–71.e15. 10.1016/j.cgh.2020.04.067 PMC8796200 32360810

[23] Vongsuvanh R, George J, McLeod D, van der Poorten D. Visceral adiposity index is not a predictor of liver histology in patients with non-alcoholic fatty liver disease. J Hepatol 2012; 57:392–8. 10.1016/j.jhep.2012.03.013 22521350

[24] Radmehr M, Homayounfar R, Djazayery A. The relationship between anthropometric indices and non-alcoholic fatty liver disease in adults: a cross-sectional study. Front Nutr 2025; 11:1494497. 10.3389/fnut.2024.1494497 PMC11747202 39839301

