# Correction to: Predicting MASLD in People With HIV Using Anthropometric Measures: A Multicenter Cross-Sectional Study

**DOI:** 10.1093/ofid/ofag586

**Published:** 2026-09-22

This is a correction to: Jihad Slim, Murad Qirem, Paul Bellafiore, Bereket Tewoldemedhin, Tala B Shahin, Kevin Leyden, Ronald Poblete, Sandhya Nagarakanti, Hugo Vargas, Predicting MASLD in People With HIV Using Anthropometric Measures: A Multicenter Cross-Sectional Study, *Open Forum Infectious Diseases*, Volume 13, Issue 5, May 2026, ofag236, https://doi.org/10.1093/ofid/ofag236

In the originally published version of this manuscript, three co-authors were erroneously listed as affiliated to “Mayo Clinic, Rochester, Minnesota” instead of “Mayo Clinic, Phoenix, Arizona”.

The corrected affiliations for these authors are as follows:

Tala Shahin, Department of Internal Medicine, Mayo Clinic, Phoenix, Arizona, USA.

Sandhya Nagarakanti, Division of Infectious Diseases, Mayo Clinic, Phoenix, Arizona, USA.

Hugo Vargas, Division of Gastroenterology & Hepatology, Mayo Clinic, Phoenix, Arizona, USA.

